# Case Report: When pain is shown and silenced. adolescents looking for a voice

**DOI:** 10.3389/frcha.2026.1829312

**Published:** 2026-05-15

**Authors:** Daniela Tortorelli

**Affiliations:** 1 Psychoterapy School, Centro Studi e Applicazione Della Psicologia Relazionale (C.S.A.P.R.), Prato, Italy; 2 Società Italiana di Psicologia e Psicoterapia Relazionale (S.I.P.P.R.), Arezzo, Italy

**Keywords:** adolescent mental health, narrative approach, self-harming behaviour, self-injury, social withdrawal, system therapy, systemic

## Abstract

Over the past two decades, adolescent psychological distress has increased markedly, with a growing number of young people experiencing intense suffering that cannot be verbalised and is instead expressed through the body, including self-harming behaviours and social withdrawal. These manifestations are frequently associated with poor therapeutic engagement and complex family dynamics characterised by emotional intrusion, overprotection, or relational absence. From a systemic and narrative perspective, symptoms are understood as meaningful communications embedded within family systems, transgenerational histories, and contemporary socio-cultural transformations. Social changes related to digital hyperconnection, altered parenting practices, and performance-oriented cultural models have reshaped adolescent subjectivity, privileging action and bodily expression over narration and mentalisation. This paper explores “shown pain” as a primary mode of communication in adolescence and examines the clinical transition from bodily enactment to narrated experience. Drawing on systemic theory, narrative psychotherapy, and neurobiological insights, the article analyses the emotional dimensions underlying self-harm and withdrawal, as well as the ambivalent regulatory functions of symptoms. A clinical case is presented to illustrate how an integrated therapeutic approach, combining individual and family therapy, can facilitate alternative narratives, support differentiation processes, and promote individual and relational change. The paper highlights the relevance of systemic psychotherapy in restoring meaning, voice, and relational listening in severe adolescent distress.

## 1 Introduction

Over the past fifteen years, youth distress has increased dramatically, becoming one of the major global health and social emergencies. In 2021, the World Health Organization stated that adolescent mental health is one of the most urgent challenges of the 21st century (1). Within this scenario, there has been a marked rise in disorders in which young people experience intense psychological pain but are unable to articulate it.

What in the 1990s primarily affected girls with anorexia and bulimia nervosa has now taken on multiple forms, extending beyond eating and nutrition disorders. Contemporary adolescent distress is increasingly expressed through phenomena such as social withdrawal, where silent renunciation becomes the embodiment of pain, and non-suicidal self-injury, where the body turns into a narrative surface, expressing a personal suffering that frequently also reflects an unspoken family drama.

Clinically, it is common to encounter adolescents who simultaneously present depressive and anxious experiences, social withdrawal, and self-harming behaviours. These are wounded and angry young people, often appearing alexithymic, who seem largely unable to ask for help and, above all, to believe that someone might truly listen. According to the latest data from ISTAT and the Guarantor for Childhood and Adolescence (2), over 50% of students report experiencing anxiety, prolonged sadness, or nervousness (51.4%), with a sharp decline in life satisfaction between the ages of 14 and 19. In 2023, psychological distress was found to affect girls more severely, with a lower psychological well-being index (67.4) compared to boys (74.3) (3). Moreover, the Lancet Commission (4) predicts a further worsening of adolescent psychological well-being by 2030. The Lancet Global Health Commission (4) highlights that approximately 75% of mental disorders develop before the age of 24, making adolescence a particularly high-risk phase.

The causes of this phenomenon are multifactorial. Social changes related to the rapid development and diffusion of digital technologies (5, 6) are identified as major contributing factors, particularly for adolescents. In Italy, the proportion of young people aged between 14 and 24 experiencing mental health problems increased from 13.8% in 2018 to 20.9% in 2022, with a particularly marked rise among females (7). Notably, a significant escalation had already occurred during the second decade of the 2000s, especially after 2010. Although the 2008 global economic crisis undoubtedly contributed to heightened levels of anxiety and uncertainty, the deterioration in young people's mental health appears to be a global phenomenon, affecting most regions of the world, albeit to a lesser extent in Asia (5). In particular, according to research by the CNR-IRPPS, Gruppo Musa (8), episodes of self-harm among girls and social withdrawal have increased substantially, the latter intensifying further after the pandemic: between 2019 and 2022, rates of social withdrawal rose from approximately 5.6% to 9.7% in just three years (9, 10).

Several authors (11, 12, 5) describe this shift as a reconfiguration of childhood. However, it may be more accurate to speak of a broader family and social reconfiguration, understood within a complex and circular framework. The pandemic acted as an accelerator of processes already underway, amplifying their social effects and generating consequences to which adaptation is inevitable, even as they become increasingly difficult to escape.

Hyperconnection, combined with increasingly fearful and overprotective parenting styles typical of the nuclear family, as well as a social climate oriented toward narcissistic recognition, has deeply altered the way relationships are formed and maintained. Young people are hyperstimulated yet emotionally disconnected as some research shows (13, 14).

The construction of adolescent identity is now heavily influenced by the constant exposure to images and short videos, which render reality unstable, fluid (15), and at times almost vaporised. Images become vehicles of meaning, while the body - or parts of it - turns into a means of communication, even for emotions that are insufficiently mentalised and therefore cannot be narrated, explained, or contained by others. Yet the development of autobiographical memory requires relational experiences, namely the presence and attentive listening of another person (16).

From a linear perspective, one might conclude that social change directly causes psychological suffering. In reality, society primarily shapes how suffering is expressed, often through behaviours that transgress socially accepted norms. Within the same social context, some individuals and families develop severe difficulties while others continue to function adequately.

Adolescents with disorders such as hikikomory or self-injury experience an intense and profound pain that they cannot translate into words or symbolise. Their voices are stifled by an inability to name emotions: narrative communication becomes paralysed. This is not a matter of emotional numbness, but rather of not knowing how to express and describe what is felt, combined with a loss of faith in the possibility of being heard and understood. These young people find themselves suspended between prescriptive family expectations and a threatening external world, unable to mentalise their emotions or legitimise their own subjectivity. Their dominant narrative (17) often takes the form of “I am crazy, a deviant among strangers” or “If I have to be the way you want me to be, I cannot do it- but I do not know how to tell you, and you would not understand, so I try not to exist”.

At the family level, parents are frequently observed to speak extensively while failing to engage with the emotional dimension or to truly listen. This scenario poses a significant clinical challenge for professionals working with these adolescents and their families: how can such pain be narrated in a way that allows it to be heard and opens up alternative possibilities? This paper seeks to contribute to this question by exploring the potential of a systemic and narrative approach in fostering verbalisation, mentalisation, and therapeutic engagement. To this end, a clinical case is presented involving an adolescent with extremely severe self-injury behaviour, in a context where family support appears deeply compromised.

## 2 Theoretical reflections

According to the systemic perspective, the symptom is a message that circulates within a meaningful system and ambivalently conveys both the need for change and the imperative to maintain the status quo of the system itself. From a complex and narrative perspective, however, the symptom also becomes a hologram of issues transmitted across generations and embedded within the social context in which the subject lives and develops, in relation to the stage of life they are experiencing with their family.

In adolescence, the symptom becomes, beyond an analogue cry for help, a symbol that defines boundaries and conveys multiple emotions. Every case that enters therapy is a subjective story that needs to be understood and made understandable. However, with those young people who are unable to put their pain into words, the first step is to listen to their emotional experience within a relationship -the therapeutic one- capable of receiving it, while attempting to reconnect those who are significant to that story, namely the family, who for countless reasons have not recognised or identified alternative ways of narrating it.

What emotions?

First of all, there is pain, manifested through the body itself, which may be used as a threat, as a canvas on which tears are written. Then there is anger, which represents the other side of pain. Like rhesus monkeys deprived of their mothers, who thrash about furiously in an attempt to find them and, when they fail, turn their aggression against themselves, this anger is initially directed toward those who are, or should be, close to these young people, and is subsequently redirected inward.

There is also the guilt of not being able to cope, which in these adolescents often turns into shame. Guilt can be atoned for, but shame remains and clings to the self, so that the socially withdrawn adolescent denies themselves to the gaze of others, fixed for years at that boundary, or the girl who cuts her skin leaves a permanent mark.

Finally, there is loneliness. When the young hikikomori locks themselves in their room, they are often not disturbed for a long time. Cuts or burns are hidden from others, or displayed without being truly seen or understood by those who should be able to recognise them. This occurs because there is an underlying pain that cannot be revealed to others for complex reasons that require exploration and understanding (18).

However, there is another feeling that contributes to the repetition of these behaviours: the relief produced by the symptom, which is linked to complex psychological, social, and neurophysiological mechanisms.

In particular, in self-injury the endogenous opioid system, the mesolimbic dopaminergic circuit and the hypothalamic - pituitary - adrenal axis are involved. The endogenous opioid system also regulates physical pain (19): some authors suggest that tissue damage stimulates the release of endorphins and enkephalins, neuropeptides with effects similar to morphine (20), producing analgesia and a subjective feeling of relaxation or relief (19, 21).

Activation of the mesolimbic dopaminergic circuit releases dopamine, producing an immediate gratification similar, albeit attenuated, to that observed in addictive behaviours. At the same time, involvement of the hypothalamic - pituitary - adrenal axis temporarily lowers cortisol levels, the stress hormone.

Alongside these mechanisms, familiar relational patterns and behaviours are activated which contribute to the repetition of the symptom. At least in the initial phase, these young people seek respite and the possibility of finding an alternative story that belongs to them - a voice of their own rather than one imposed or desired by others to whom it seems impossible to say no. They use their bodies to do so: their defence thus becomes both their prison and the prison of the family members involved.

## 3 Clinical case

Lucia^1^ is 19 years old. Her mother, Mery, calls me literally begging for an appointment. She is extremely anxious and, already during the phone call, she declares “she's crazy, she's been divergent since birth… she does it to detach herself from me.” as if her daughter's behaviour were motivated by spite. Amid the abundance of information, the element that strikes me is this last sentence from Mery, which points to a repeated narrative that co-constructs a consolidated dominant reality (17), one that I imagine will be difficult to dismantle. I had no immediate availability and scheduled the appointment after a long wait, which the family nevertheless accepted.

Lucia arrives dragged in by her parents, Mery and Gianni, half asleep and heavily medicated. She is overweight -the family confirms that this is linked to the use of certain psychiatric drugs-, has multicoloured hair, appears neglected, and is covered in piercings, cuts, and burns - on her arms, body, legs, and face.

Lucia has already dropped out of two previous individual therapeutic experiences, both interrupted due to poor compliance and because, as she says, “they didn't give me anything!”. Parents say that she has attempted suicide three times, at ages 15, 18, and 19, the last occurring only a few months prior to consultation. At the time of our assessment, she is followed only by a psychiatrist who has diagnosed borderline personality disorder (BPD; DSM-5 (22);; code F60.3), with severe self-harming behaviour and prescribed significant doses of mood stabilisers, benzodiazepines, and antidepressants. The diagnosis was also confirmed through a systemic-relational assessment, grounded in the information collected and in the observation of behavioural patterns and relational interactions.

She sleeps with her mother, who never leaves her alone, while Lucia's bedroom is permanently locked. The house is filled with cameras monitoring the girl's every movement. Her father, a very busy man with an important role in the banking sector and a Harvard graduate, appears distant and peripheral: emotionally cold, ironic, and often sarcastic. Her mother, a part-time teacher, seems exhausted, spitting venom and anguish, oscillating between attacks and self-pity. Her entire life is organised around Lucia - telling her what to do and how to do it, about everything, as always. The only university graduate in her family of origin, she left a promising job at a major company to care for her children, while her husband pursued the “great career”.

Lucia has an older brother who is following in his father's footsteps, considered “normal”, successfully studying economics and suffering from panic attacks. During the joint consultation, it becomes immediately clear that the father occupies a peripheral position and that there is a marked emotional distance between him and Lucia. He works extensively and, when he returns home, isolates himself in the garage, absorbed in his hobbies; occasionally he explodes when the shouting and arguments between Mery and Lucia exceed a certain threshold. With her mother, by contrast, Lucia's involvement appears maximal, to the point of suggesting a symbiotic relationship. I also notice that Lucia seems deeply irritated by her mother and tends to speak very little, likely also due to the medication she is taking. She repeatedly emphasises: “They are crazy, not me. I just want to be left alone. Everything I do is wrong… it always has been!”. Lucia is enrolled in a short university programme, strictly chosen by her mother, which she does not attend.

Her brother, although invited, does not attend the session.

## 4 Taking charge and structuring the setting

The preliminary consultation phase allows for the exploration of hypotheses and meanings and should facilitate the construction of shared understandings, the assessment of expectations and concerns, and the decision regarding the most appropriate therapeutic setting (23).

After the first session, I state that the *conditio sine qua non* for my decision to take Lucia into treatment is the activation of family therapy in parallel with individual work, as this form of suffering involves the entire family system, which represents a crucial resource. This decision was also guided by the clinical aim of promoting an initial differentiation between Lucia and her mother, and of re-establishing clearer boundaries between the parental and child subsystems (24, 25).

The parents accept this proposal, almost surprised, both because their focus is entirely centred on Lucia and because I succeed in involving the father, who had previously remained completely peripheral. I notice a satisfied yet bitter smile on Lucia's face. The girl was positively surprised by a contextual and relational diagnostic approach that seeks meaning in the distress and framed the parents' involvement as a resource.

The family agrees to undertake family therapy at the Centre for the Study and Application of Relational Therapy and individual therapy for Lucia with myself, psychotherapist specializing in systemic-relational therapy.

### 4.1 The pain shown and the voice of lucia

When I first see Lucia on her own, she looks at me with defiance. She says she has never understood what happened; she only knows that her parents are “emotionally illiterate”.

She has always loved animals and drawing. She is exceptionally intelligent, but her educational trajectory has nonetheless been experienced as a torment. At secondary school she was compelled to enrol in the scientific lyceum, in line with the performance-driven expectations typical of her family. Shortly thereafter, she began to self-harm by cutting and, despite achieving very high marks, she failed the year due to behavioural concerns and repeated absences. She was subsequently transferred to the classical lyceum. The cutting intensified and she was admitted to hospital on two occasions following suicide attempts. The first time, she ingested a large quantity of benzodiazepines; the second time, she inflicted deep cuts upon herself in order to be re-admitted and to rest. On both occasions she was discharged with substantial pharmacological prescriptions.

As the years passed, her condition deteriorated. Only in the fourth year of secondary school was she finally allowed to attend the art lyceum - the school she had wished to attend from the outset, but which had never been permitted, as her parents considered it useless. She achieved excellent results there; meanwhile, however, her life was unravelling. Self-harm (to legs, hips, arms and face), a third hospitalisation for alleged attempted suicide, experiences of bullying, profound relational difficulties, excessive mobile phone use, increasing isolation, and eventually a prohibition on attending her beloved horses.

Engagement in individual sessions occurred primarily through attention to emotions, not least because Lucia spoke very little. She recounted her cutting and hospital admissions and stated: “I didn't want to die, but I pushed into my flesh in order to be admitted. In hospital, they let me do what I wanted. They didn't ask anything of me. Everyone was kind and I could breathe a little.” She showed me the worst cut: vertical, on her wrist, searching for the vein - twelve stitches.

I narrowed my eyes and sighed: “What pain!”.

She smiled, proud: “Pain? No. No pain. I like it.”

And I, surprised: “I didn't mean here - the wrist - but here - the heart.”

Her eyes filled with tears.

Lucia will need to articulate a great deal of anger and pain particularly in relation to the father, whose emotional distance and apparent delegation of both educational and affective functions, as well as in relation to the mother who, since Lucia's early childhood, has insulted her and criticised her with extraordinary ferocity, only to weep afterwards and insist that everything she has done has been for her children.

### 4.2 Joint and parallel family and individual treatment

In the initial phase of joint treatment is marked by frequent and intense family conflict, but Lucia begins to show signs of improvement.

Family therapy quickly becomes a battlefield: the more Lucia improves in terms of symptomatology and acquired and maintained autonomy, the more fiercely her mother attacks - her daughter, the family therapy itself, and me - particularly when Lucia begins to legitimise her own choices. This pattern suggested difficulty in accepting her daughter's emerging individuality. At the same time, the mother continued to rely on therapeutic interventions, displaying marked emotional ambivalence.

At this stage, the father does not yet know how to withdraw from the situation, but he begins to understand that he can take an active role. He moderates his sarcasm toward his daughter and starts to support her in practical ways, for instance by helping her with driving lessons. At times, he also has to confront Mery directly, acting as a protective shield for Lucia.

Meanwhile, individual therapy enables work with Lucia through the construction of a different narrative of her pain, which gradually begins to be expressed and heard. At the same time, the first steps toward autonomy emerge: the cut starts to transform as Lucia's real differentiation from her mother. Lucia stops cutting herself; it is agreed that her room will be reopened, and she begins to go out again with a small group of friends.

More recently, Lucia recounts a memory from nursery school. She was very young, and her mother had loosened the cap on her water bottle to make it easier for her to open during the morning. When Lucia entered the classroom, she noticed that the water had spilled because the cap had been screwed back on incorrectly. She cried all morning - not out of fear of her mother's reaction, but because she was terrified of having ruined a rare gesture of affection from her mother.

Family therapy lasts six months and ends when Mery, supported by Gianni, declares that she does not feel understood by the therapeutic team, whom she had attacked fiercely since the very first meeting (initially because the psychotherapist had not welcomed her properly at the door). The messages sent are destructive and pleading, revealing severely polarised thinking. Nevertheless, the time spent in therapy proves useful in bringing about certain structural changes (24), which help to detriangulate Lucia and shift greater responsibility onto Gianni.

Lucia's individual psychotherapy, by contrast, continues.

The parents continued to support Lucia's individual therapy financially and to engage in scheduled telephone contacts with the therapist, in agreement with their daughter. These aspects reflect a high level of treatment compliance. Over the course of treatment, they also conveyed explicit appreciation for the improvements observed, particularly in the final phase of the intervention.

The father's tentative rapprochement primarily reinforces the alternative narrative (17), which no longer portrays Lucia as a rebellious daughter intent on destroying her mother, but as a highly intelligent young woman endowed with important characteristics that her parents themselves have transmitted to her. She is rigorous, punctual, reliable - traits that belong to the family's semantic field, though not necessarily positioned on opposite poles (26): functional qualities that Lucia can now use in the service of a project she has chosen for herself.

### 4.3 The narrated pain

The transition from shown pain to narrated pain represents a crucial turning point in the therapeutic process with adolescents who use their bodies as their primary means of communication. In Lucia's case, self-harming behaviour had long functioned as the only possible language—powerful and deeply ambivalent - capable of providing temporary relief from emotional overload and a momentary respite from relentless demands and attacks, while simultaneously conveying a sense of control that ultimately became a form of imprisonment.

The body becomes the hologram of what cannot be thought or spoken. Self-harm can thus be understood as a reappropriation of the self when the *other* fails in their function of recognition. In Lucia's case, as in many similar clinical situations, the *other* is the mother, who, under the guise of care and sacrifice, exercises a psychological intrusion as heavy as abuse. The *other* is also an emotionally and physically absent father who, in order to avoid conflict or out of convenience, fails to provide sufficient ethical support for his daughter, leaving her - perhaps in good faith - at the mercy of the parent deemed most suitable for her upbringing: the mother-teacher (27). Within this relational configuration, separation and individuation are rendered unthinkable.

From a systemic perspective, a stalemate emerges (28): within the mother's delusional organisation, the husband effectively does not exist, as he is absent from her psychic world. Mery relinquishes her career to Gianni but appropriates Lucia. Gianni, in turn, fails to claim his paternal role. Lucia protests without arguments and, through the ambivalence of the symptom, simultaneously withdraws from and prescribes a synchronic relationship, positioning herself between two parents who have likely not functioned as a couple for some time. Meanwhile, Nicola (the brother) slips away quietly, almost fearfully.

A positive interpretation in terms of Paradosso e Controparadosso (29) might suggest that Lucia is sacrificing herself to keep the family together, avoiding confrontation within the parental couple and freeing her brother. Clearly, no one asked her to do so. Such an interpretation, although powerful, could have provoked the mother's rage and would not have afforded Lucia the time she needed to be heard.

These mothers - often imperious bearers of profound psychological distress- are perceived as impossible to confront, as they are considered too fragile to withstand criticism or separation. Unable to oppose Mery, Lucia turns the aggression against herself. She longs to separate but cannot; deprived of words at the point where she is wounded as a person, she speaks through action, tormenting her own skin. The regular, geometric cuts evoke a need to impose order on herself and her thoughts. Tattoos fix fragments of identity; the colours of her hair illuminate the darkness of her life; piercings become the recognition she never received.

Individual sessions thus become densely meaningful.

Parallel family work initiates movement at a relational level, creating the time and space for action to be transformed into words. When pain is accepted and legitimised within the therapeutic relationship, the body can gradually be relieved of its role as spokesperson. Narrative and systemic therapy does not erase pain, but it renders it shareable and therefore thinkable. Ideally, the therapeutic relationship becomes a bridge that fosters listening within and between significant relationships, particularly within the family. This is more readily achievable when initial resources are present; otherwise, such resources must be co-constructed.

Relational psychotherapy with the individual requires the presence of the *other* - if not physically in the room, then certainly within the therapist's mind (30).

### 4.4 Follow up

Today, after two years of treatment, Lucia is continuing individual therapy, with her parents'support. She has discontinued all medication, lost weight, adopted a single hair colour, and reduced her piercings to two. She has obtained her driving licence and works in a highly prestigious bar, where she is greatly valued, partly because she speaks excellent English and Spanish. She has successfully completed a bartending course, purchased a car, and is planning to enrol at university. Despite her young age, she lives independently and maintains a stable relationship with her boyfriend.

Relation between Lucia and her parents have improved significantly: in particular, there are fewer arguments between mother and daughter and there is greater mutual understanding and acceptance of each other's differences.

Mery has begun individual psychotherapy, which has proved highly beneficial. Gianni continues to support both women through small yet significant contributions. Lucia's brother lives three hundred kilometres away from the family of origin.

The construction of an alternative narrative- one that no longer defines Lucia as “divergent” but as a strong-willed, precise, and capable young woman who is painstakingly building herself in her own way - has allowed for a profound reorganisation of her identity, an ongoing process that Lucia herself recognises still requires work.

At this stage, following a long and not always easy process, and having been emotionally legitimised, Lucia can cautiously begin to understand not only herself but also the limitations and unresolved pains of her parents. In particular, she comes to grasp her mother's history: growing up with a gambling, violent father and a submissive, dependent mother whom Mery could neither question nor oppose, but only please. Giving up her own career brings Mery dangerously close to her mother's intolerable failure and triggers a mechanism of projective identification toward her daughter, who must redeem her mother at all costs, without ever being allowed to leave or move on.

Gianni, raised within an extremely high-achieving and patriarchal family, willingly accepts the parental and marital mandate to build a prestigious career that ensures economic security for the family.

## 5 Conclusions and research perspectives

The clinical case presented can be considered an example of how many forms of contemporary adolescent distress are expressed through the body even before words emerge. In social and family contexts where emotional expression is impoverished or distorted, shown pain may assume the function of an extreme form of communication, consistent with the subject's relational and emotional history. Reducing such manifestations to dysfunctional behaviours or diagnostic labels risks obscuring their meaning, reinforcing isolation, and contributing to chronic outcomes.

It is therefore useful to restore complexity and voice to these stories, recognising how individual, familial, and socio-cultural factors intertwine to sustain dominant, dysfunctional, and painful realities. In severe cases, beginning in adolescence, the integration of individual and family settings - where possible - allows simultaneous work on legitimising the young person's self and reorganising the relational dynamics that maintain the symptom, thereby facilitating healing at both individual and systemic levels.

Whenever possible, it is advisable to recover the listening capacity of the family of origin even more than that of the individual therapist. When this is not feasible, the therapeutic alliance itself may offer the young person a vital opportunity. Relationships cannot always be repaired, but this possibility should never be abandoned by the systemic therapist without genuine attempts.

From a clinical standpoint, the therapist has to cultivate deep listening, patience, and the ability to suspend premature restructuring interventions - an especially demanding task for systemic therapists working in emergency contexts. Careful attention to timing is essential, as these young people must first learn to narrate.

Only after a process of legitimisation do more articulated narratives often emerge - sometimes unexpectedly - including transgenerational stories that are crucial for understanding the origins of emotional incapacity. Such understanding allows the adolescent to preserve the parental image from distorted emotional functioning without falling into justification or blame. An alternative narrative that explains the source of pain enables anger to diminish or dissipate.

Finally, it is essential to reflect on the emotions evoked in the therapist which, if unprocessed, may compromise listening and lead to premature intervention. Shown pain is powerful and distressing; in emergencies, the impulse is to act rather than to listen. The therapist have to learn to inhabit this narrow space and attempt to build bridges with those who are most needed: the family.

Only later can these young people begin to understand their parents and the pain the parents themselves have denied - when their bodies and selves are sufficiently intact and differentiated to allow such understanding.

From a research perspective, further investigation is needed into the links between social transformations, family configurations, and bodily expressions of distress in adolescents. Longitudinal studies integrating clinical, neurobiological, and systemic data would be particularly valuable in clarifying how unmentalised pain translates into action on the body. Comparative research on the effectiveness of different intervention models, especially integrated approaches, is also warranted.

Systemic therapy may offers a framework for understanding, meaning-making, and relational repair. The presence of parents may allow for reparation (31, 18); when this is not possible, their symbolic presence within the therapist's mind and within the narrative process still allows for understanding.

## Data Availability

The raw data supporting the conclusions of this article will be made available by the authors, without undue reservation.

## Footnotes

^1^All personal references have been appropriately modified.
